# When Bitcoin encounters information in an online forum: Using text mining to analyse user opinions and predict value fluctuation

**DOI:** 10.1371/journal.pone.0177630

**Published:** 2017-05-12

**Authors:** Young Bin Kim, Jurim Lee, Nuri Park, Jaegul Choo, Jong-Hyun Kim, Chang Hun Kim

**Affiliations:** 1 Interdisciplinary Program in Visual Information Processing, Korea University, Seoul, Korea; 2 Department of Computer Science, Korea University, Seoul, Korea; 3 Department of Software Application, Kangnam University, Yongin, Korea; University of Texas at San Antonio, UNITED STATES

## Abstract

Bitcoin is an online currency that is used worldwide to make online payments. It has consequently become an investment vehicle in itself and is traded in a way similar to other open currencies. The ability to predict the price fluctuation of Bitcoin would therefore facilitate future investment and payment decisions. In order to predict the price fluctuation of Bitcoin, we analyse the comments posted in the Bitcoin online forum. Unlike most research on Bitcoin-related online forums, which is limited to simple sentiment analysis and does not pay sufficient attention to note-worthy user comments, our approach involved extracting keywords from Bitcoin-related user comments posted on the online forum with the aim of analytically predicting the price and extent of transaction fluctuation of the currency. The effectiveness of the proposed method is validated based on Bitcoin online forum data ranging over a period of 2.8 years from December 2013 to September 2016.

## Introduction

The advancement of the ubiquitous Internet has resulted in the emergence of unprecedented types of currencies that are distinct from the established currency system. The rise of these so-called cryptocurrencies, of which the total supply is increased by using a unique method known as “mining”, has changed the way economic transactions are conducted among Internet users to a great extent. Following the introduction of Bitcoin in 2008[1], a range of cryptocurrencies comparable to Bitcoin have come into existence since 2010[2–4]. Currently, Bitcoin and other cryptocurrency variants are often used for online payments and transactions[4–6] with their circulation gradually increasing over time[3, 6].

In parallel with the increasing circulation of Bitcoin, a growing number of Bitcoin users take to social media or online Bitcoin forums to share information[6]. Yet, despite the plethora of information posted by Bitcoin users, the linkage between such postings and Bitcoin transactions has not been well-documented.

The present research builds on previous findings regarding Bitcoin-related online forums, and proposes a method to analytically predict the fluctuations in Bitcoin transaction counts and value using the data collected from user comments posted on the online forum. First, we extracted keywords of interest from user comments on the online forum. We analysed the relationship between the Bitcoin transaction count and price based on the extracted keywords and quantification. Then, we developed a model based on deep learning[7, 8] to predict the Bitcoin transaction count and price. The proposed method efficiently processed the readily accessible online data, and identified as well as utilized the elements that online forum users perceived as important.

## Related work

Research on cryptocurrencies, particularly on Bitcoin, has been extensively conducted from diverse perspectives, e.g. the analysis of user sentiment as manifested by social media including Twitter[9, 10]. The aim is to determine the value of Bitcoin relative to social phenomena and incidents that have taken place since the introduction of the currency. These social phenomena and incidents include research on the extent to which Bitcoin price fluctuations are related to web search query volumes on Google Trend and Wikipedia, i.e. the extent to which these query volumes predict the Bitcoin price and trade volume[11–14].

Some recent research has focused on the characteristics of Bitcoin online forums. People who share common interests tend to post comments concerning certain topics on online forums[15–19]. Bitcoin is mostly traded on the web with many users making buying/selling decisions based on information acquired on the Internet[6, 20]. Therefore, it is possible to observe how users respond to daily Bitcoin price fluctuations, and to identify or predict future fluctuations in the Bitcoin price and trade volume [6, 20]. In addition, forum users are analysed and classified into Bitcoin user groups[6].

Some researchers simply analysed sentiments based on comments posted by forum users or focused on users per se without considering the information derived from cumulative user comment data gathered during a sample period[17, 21, 22], while others analysed online user comments.

In this regard, topic modelling has been actively explored as an effective technique for analysing user opinions from their online textual postings[23]. Topic modelling[24, 25] is a text-mining technique that extracts a set of prevailing topics and relevant keywords out of a large-scale document corpus. This topical information provides users with an instant overview of the corpus, thereby obviating the need to read through comments, which would otherwise be a tedious, time-consuming process.

Recently, collaborative filtering and topic modelling have been integrated for generating scientific article recommendation systems on an online community[26]. A Temporal Latent Dirichlet Allocation (TM-LDA) system was used to conduct an in-depth analysis of the online social community by employing an advanced Latent Dirichlet Allocation (LDA) topic modelling algorithm[27]. Likewise, application of the LDA approach to Chinese social reviews revealed the sentiments underlying some social events and services[28].

## Methods

### System overview

**Fig 1:**
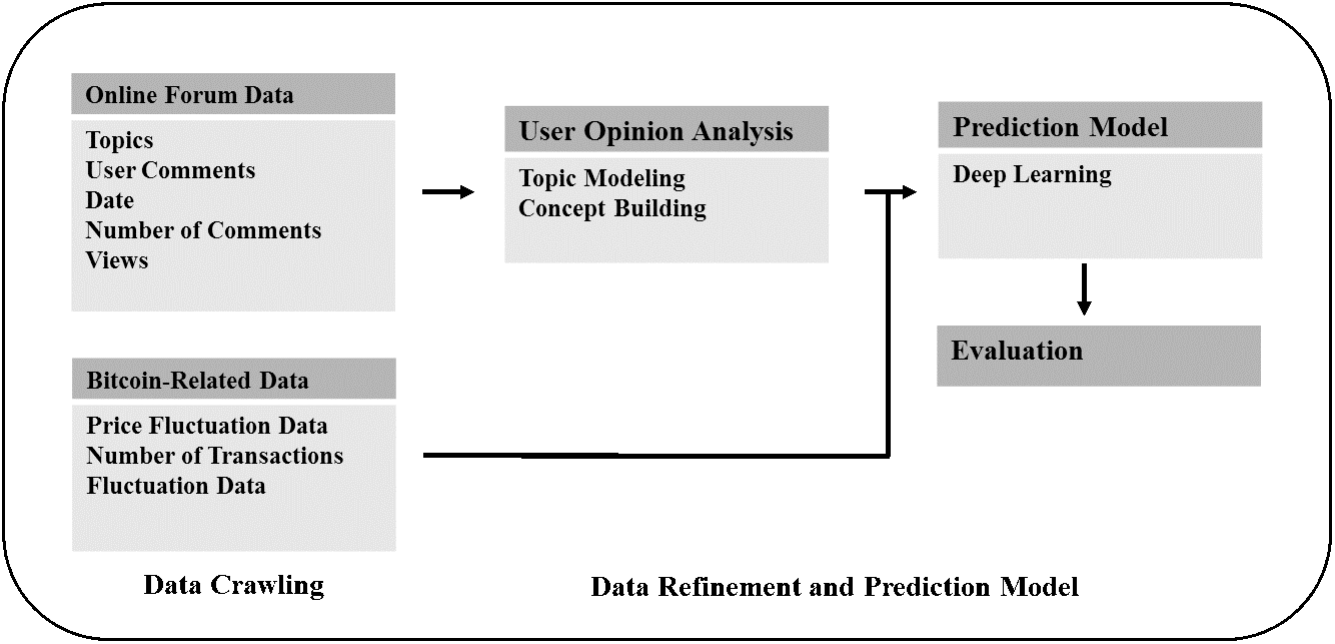
System overview.

This section provides an overview of the proposed method. First, we gathered the data relevant to Bitcoin for the purpose of the experiment. More specifically, Bitcoin-related posts on the online forum, daily Bitcoin transaction counts, and its price were gathered. We also extracted and rated significant keywords from the data gathered on the online forum. Then, we selected the data of higher score ratings to generate the prediction model based on deep learning and used the model to predict the fluctuation in the Bitcoin price and transaction count (see Fig 1).

### Data crawling

Data crawling was the first step in our analysis. The online environment for Bitcoin transactions is well defined and the rise/fall in its price depends on the supply and demand arising from users [2, 3, 5, 6]. We postulated that user comments on the targeted online Bitcoin forum would have an impact on the fluctuation of the Bitcoin price and transaction count. Thus, we crawled and analysed the relevant data.

The large online forum is home to a variety of Bitcoin-related topics, where users actively engage in conversations by posting comments and forming threads[6, 29]. The bulletin boards on the Bitcoin online forum are largely comprised of four different sections. Each section consists of three to five sub-sections. For example, the ‘Bitcoin’ section is sub-divided into ‘Development & Technical Discussion’, ‘Mining’, ‘Bitcoin Discussion’, ‘Project Development’, and ‘Technical Support’. We crawled the ‘Bitcoin Discussion’ subsection under the ‘Bitcoin’ section where comments are posted most actively.

The threads of comments and replies posted from 1 December 2013, when Bitcoin started to sweep the globe, until 21 September 2016 were crawled. Each thread, including the topics and all relevant replies, the time when such posts appeared on the forum, the number of replies posted, and view counts were crawled as well. Duplicate sentences were removed from the replies that quoted earlier posts or replies prior to crawling. We collected data in a legitimate manner, in compliance with the terms and conditions. Moreover, the collected data did not involve any personally identifiable information. The.json files of the Bitcoin forums crawled are presented in the Supporting Information.

Furthermore, we used Coindesk to crawl the daily Bitcoin price and the number of transactions for the abovementioned sample period (See Table 1).

**Table 1:** Summary of crawled data.

Opinion Topics		
Crawling Source	Crawling Boundary	Data Volume
Bitcoin Forum (https://bitcointalk.org)	Dec. 01, 2013~ Sep. 21, 2016	17,381 forum articles, 627,122 user comments
CoinDesk (Bitcoin Prices and Transactions)	Dec. 01, 2013~ Sep. 21, 2016	1,026 Prices and Transactions Value (1 value per day)
Google Trends (Bitcoin)	Dec. 01, 2013~ Sep. 21, 2016	1,026 Google Trends Values (1 value per day)
Wikipedia Usage (Bitcoin)	Dec. 01, 2013~ Sep. 21, 2016	1,026 Wikipedia Usage Values (1 value per day)

In addition, we reinforced the learning model by crawling the widely used Google Trend data and Wikipedia usage data. Google Trend shows the search interest in a certain keyword on a scale of 1 to 100 based on its search volume on Google for a certain sample period. Google Trend data is widely used to analyse data and phenomena in multiple disciplines[30–34]. We gathered Google Trend data related to the keyword “Bitcoin”. The Wikipedia usage volume data is based on the page views of a certain keyword on a certain day, and broadly used in many analytical studies on data or Internet phenomena[34–36]. Again, we gathered data about the keyword “Bitcoin” on Wikipedia. Table 1 outlines the arrangement of opinion and market data crawled.

### Analysis of user comment data

Our intention was to extract significant keywords used in Bitcoin transactions from the aforementioned crawled data. Therefore, we conducted topic modelling on every user comment to extract the keywords, which were in turn subjected to kernel density estimation for score rating.

#### Concept building

**Fig 2:**
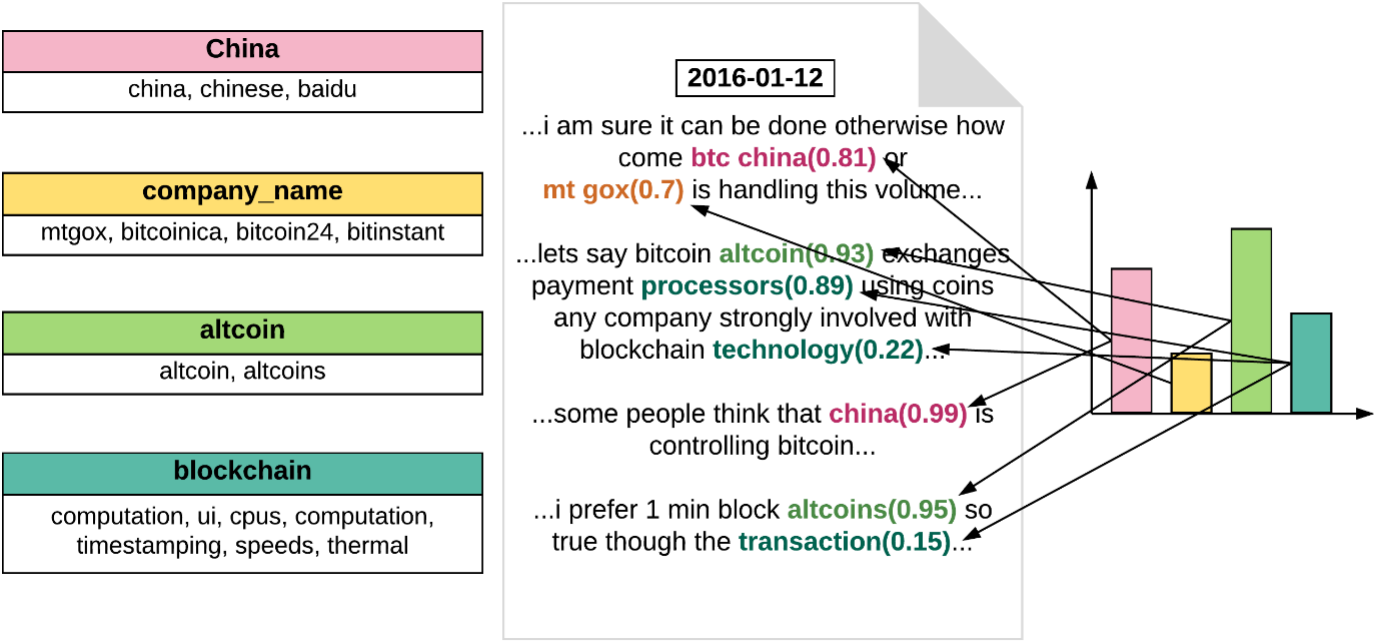
Concept for building the workflow.

Our main goal was to extract quantitative features related to diverse characteristics from documents (see Fig 2). We considered the feature value as the degree of relevance for a feature. In detail, the feature value represents the extent to which a document has a particular characteristic. For example, sentiment analysis concerns one such quantitative feature, or the extent to which a document is positive or negative. We generalised this idea to various other user-defined characteristics. Examples of such characteristics include the extent to which a document is related to finance, immigration, and family issues. In particular, we built a lexicon, i.e. a set of keywords, relevant to the characteristics and utilised it to assign a feature value to a document by computing the degree to which the document contains those characteristics defined in the lexicon and other potentially relevant keywords. In this study, we considered a characteristic to be a concept describing a particular phenomenon or object, and defined a concept by constructing a set of keywords, whose meanings were relevant.

Concepts can play an important role in document analysis in diverse fields. That is, one can build useful domain-specific concepts in economics, politics, and social sciences and define the characteristics of documents with respect to these concepts. In the case of a spam-filtering task on documents and comments, for example, we can actively employ a ‘spam’ concept consisting of suspicious terms that usually appear in spam mails to measure the likelihood of the comment being unsolicited mail.

Here, the concept building process was composed of two steps: (1) the initial construction of a relevant keyword set, followed by its (2) user-interactive expansion. In order to facilitate the first step, we provided a user with the initial sets of coherent keywords obtained with two different techniques. The first technique we used was topic modelling, which algorithmically computes those representative keywords emerging from a document corpus. The user can then select some of them as an initial word set for their own concepts. As the other method to provide initial keywords, we computed the representative keywords from the centroid vectors obtained by k-means clustering on word embedding vectors[37].

Once a user formed an initial, small-sized lexicon for a particular concept, the second step was to interactively expand it by using a recently proposed visual analytics system named ConceptVector. Based on the initial lexicon given as user inputs, ConceptVector recommended potentially relevant keywords to enable users to easily add a subset of them to the lexicon. As the lexicon expanded, ConceptVector adjusted the recommended keywords that match the semantic meaning of the concept.

The foregoing procedure is discussed further below.

#### Topic modelling for initial lexicon building

The topic modelling approach we used to extract representative keywords emerging from a document corpus is non-negative matrix factorisation, where the non-negativity allows users to interpret the value from factor matrices as the relevance score of a word or a document to a particular topic as mentioned above.

In particular, we constructed a document-term matrix *A* from the 17,381 forum articles and 627,122 user comments collected from the Bitcoin forum (See Table 1). Each article contains five attributes, ‘content’, ‘topic’, ‘comments’, ‘date’, and ‘views’, whereas each comment contains ‘content’ and ‘date’ features. Using the `date’ field, we split the document-term matrix per day for our analysis. We then applied the topic modelling to each so as to extract the different topic sets and their representative keywords across different dates.

The mathematical details of this process are as follows. Given a document-term matrix $A\in R^{m\times n}$ where *m* is the number of articles and *n* is the dictionary size, Non-negative Matrix Factorization(NMF) approximately factorises it into two matrices $W\in R^{m\times d}$ and $H\in R^{d\times n}$, where *d* represents the number of topics (50 in our study), e.g.

$$\min_{W,H}||V-WH|{|}_{F},\;\mathrm{where}\;W\geq 0,H\geq 0$$ (1)

The columns in the resulting matrix W correspond to different topics and the keywords corresponding to the dimensions of the *k* largest value in each column function as the representative keywords of the topic.

#### Expanding the lexicon via word recommendation

We proposed two types of concepts in the system. A unipolar concept represents exactly one concept such as crude oil and immigration. A bipolar concept has two polarities that oppose each other, e.g. positive vs. negative, progressivism vs. conservatism. In the case of building a concept, the system has positive, negative, and irrelevant word sets. When a user provides a word as an input, the system provides 50 recommended words that are potentially relevant to the seed word. We then automatically sorted the recommended words into five clusters, using the *k*-means clustering, to gather closely related terms into one group.

Once the lexicon of a concept is created by user interactions, the document rating process utilises the concept built in the process above. Because of the lack of expression resulting from the limited number of words a person could manage, we applied the kernel density estimation (KDE) in the word rearranging phase.

#### Computation of document relevance to concept

Prior to the KDE, the concept had a limited number of descriptive terms for a characteristic, which resulted in a lack of expression and description. Therefore, the KDE served for the probabilistic smoothing over every word. This smoothing process is the most important procedure for document analysis since the score rating process cannot consider synonyms or closely related words that also represent a specific concept. Based on the assumption that the input terms describe the concept sufficiently well, we constructed a kernel that exerts influence on the entire vocabulary. ConceptVector adopts a Gaussian kernel as described below.

For the class *y* ∈ {*positive,negative,irrelevant*}, the conditional probability for each class can be calculated by the distance function *d* that represents the distance between a word in the word set in each class and the kernel *k* that ensures a proper balance between the given word and the others. The conditional probability of a keyword *z* for a class *c* can be computed as below:

$$p(z|y=c)=\frac{1}{\#(x\in c)}\sum_{x_{i}\in c}k(d(x_{i},z))$$ (2)

which can also be seen as the relevance score to each class.

Since our final goal was to obtain scores by taking all classes into consideration, we rated a concept in view of all classes. For instance, ‘happy’, in the case of a bipolar concept, was rated for the positive, negative, and irrelevant classes. We calculated the bipolar rating as below:

biscore(z)=rel(z)∙{p(y=positive,z)−p(y=negative,z)} (3)

rel(z)=1−p(y=irrelevant|z) (4)

The range of the bipolar score is [-1, 1] because the max value of *p*(*y* = *positive,z*) and *p*(*y* = *negative,z*) are 1.

### Prediction modelling

#### Granger causality test

The Granger causality test is based on the supposition that if a variable X causes Y, then any change in X will methodically happen before any change in Y[17, 22, 38]. As shown in past research, slacked estimations of X display a measurably noteworthy connection with Y[17, 22, 38]. Nevertheless, connection does not imply causation. We test whether the time arrangement of a discussion of conclusions contains any prescient data with respect to vacillations in the Bitcoin transaction and price.

Our time arrangement at the Bitcoin transaction count and price, indicated by *S*_*t*_, reflects day-to-day change in the Bitcoin transaction count and price. To test whether the idea of gathering feelings in the time arrangement could forecast the change in the vacillation in terms of the Bitcoin transaction and price, we considered the difference clarified by two linear models as in (5) and (6) below. The first model uses just *n* slacked estimations of *S*_*t*_ for the forecast. However, the second model uses the *n* slacked estimations of both *S*_*t*_ and the time series of a concept of forum opinions, meant by *X*_*t*−1_,⋯,*X*_*t*−*n*_. We completed the Granger causality test as indicated by the models in (5) and (6).

$$S_{t}=\alpha +\sum_{i=1}^{n}\beta_{i}S_{t-i}+\epsilon_{t}$$ (5)

$$S_{t}=\alpha +\sum_{i=1}^{n}\beta_{i}S_{t-i}+\sum_{i=1}^{n}\gamma_{i}X_{t-i}+\epsilon_{t}$$ (6)

In view of the consequences of the Granger causality test, we can reject the null hypothesis, whereby the time series of a concept of forum opinions does not predict fluctuations in the Bitcoin transaction count and price with a high level of confidence. The Granger causality test was performed on the Bitcoin transaction count and price for a time lag of 1 to 12 days.

#### Deep learning model

Using the gathered data and the analysed and rated comment data, we built a model for predicting the fluctuation in the Bitcoin price and transaction through deep learning. Deep learning is widely used for addressing diverse challenges[8, 39]. Despite the quantitative and qualitative increases in Bitcoin-related formal and informal data following the broadening applicability of Bitcoin, deep learning has rarely been used to explore Bitcoin price trends and to address other Bitcoin-related challenges. We created a setting to apply deep learning to the data spanning a period of 2.8 years.

As the first step, we standardised the data to improve its applicability to the learning model. An example of applicable input data is provided in Table 2.

**Table 2:** Example of deep learning data set. The z-score ($Z_{E_{t}}=\frac{E-\bar{x}(E)}{\sigma (E)}$, where $\bar{x}(E)$ and σ(E) represent the mean and standard deviation for every date, respectively) of data for the previous 12 days (*t* = 12) was used as the values.

Data Class	Date	KDE-Based Concept Scoring Data										Formal Data				
		Concept										Number of Topics	Sum of Replies	Sum of Views	Google Trend Value	Wikipedia Page Views
		1	2	3	4	5	6	7	8	9	10					
Crawled and Analysed Data	1 Sep. 2016	*A*	*B*	*C*	*D*	*E*	*F*	*G*	*H*	*I*	*J*	*V*	*W*	*X*	*Y*	*Z*
Input Learning Data	2 Sep. 2016	$Z_{A_{t}}$	$Z_{B_{t}}$	$Z_{C_{t}}$	$Z_{D_{t}}$	$Z_{E_{t}}$	$Z_{F_{t}}$	$Z_{G_{t}}$	$Z_{H_{t}}$	$Z_{I_{t}}$	$Z_{J_{t}}$	$Z_{V_{t}}$	$Z_{W_{t}}$	$Z_{X_{t}}$	$Z_{Y_{t}}$	$Z_{Z_{t}}$

Subsequently, to use the input data for prediction, we set up a deep learning model. Multiple hidden layers were accumulated for learning to identify deep data structures. Specifically, 1, 2, 3, and 5 hidden layers were constructed to select the layer structure that returned the best possible prediction result. The number of neurons that were allocated to each hidden layer was 1,024.

As for the input layers, based on the input data provided in Table 2, 15 input data points were represented as serial vectors to allocate neurons based on the cumulative number of days spent on learning, i.e. 45, 75, 105, and 180 neurons were allocated to cumulative 3, 5, 7, and 12 days. As for the output layer, two neurons were allocated while the probability of rise/fall was represented with the softmax function. The prediction model was built using Google Tensorflow[7], and GPU operation (nVIDIA CUDA) was used to accelerate the deep learning process.

## Results

### Concept building results

**Fig 3:**
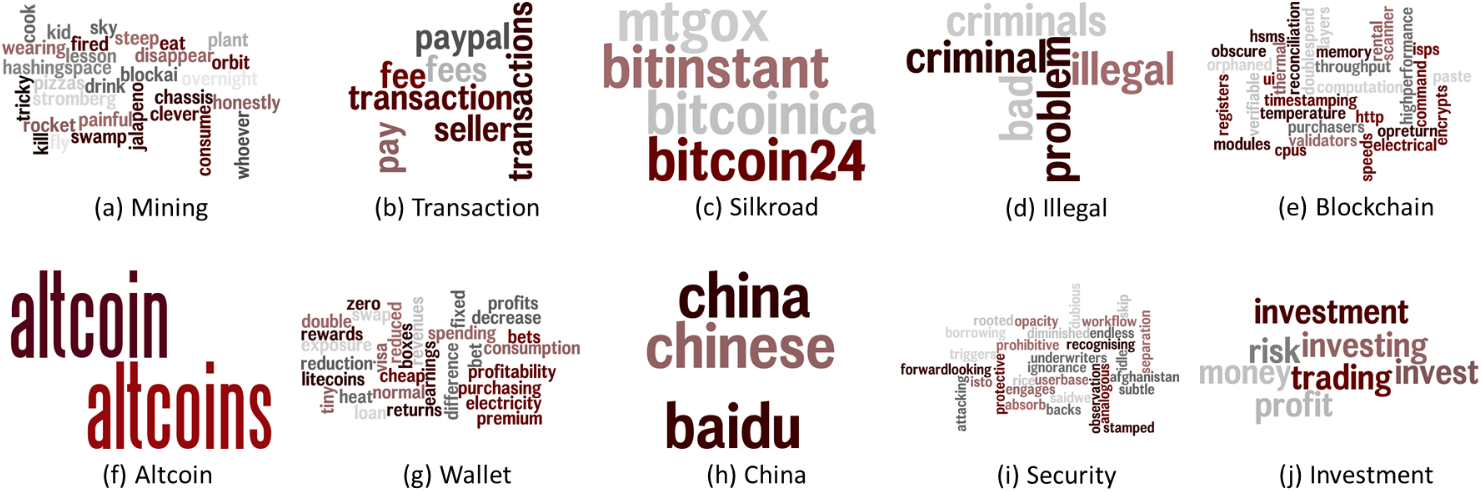
Ten topics generated by the Bitcoin forum documents.

Fig 3 shows the concept derived from the concept building phase and the words constituting the concept. We focused on a general phenomenal analysis of the meanings of the concept, rather than analysing all the words constituting the concept.

Because mining is a means of earning Bitcoin, many users share their opinions about its efficiency. In addition, the fundamental algorithm by which the Bitcoin is operated, namely ‘blockchain,’ is often discussed. Other than mining, Bitcoin can also be earned by transactions. Therefore, it is possible to conduct transactions with investment character, in which case related concepts include ‘transaction’ and ‘investment’. Moreover, the ‘wallet’, a kind of repository in which Bitcoin can be stored and used in subsequent transactions via mining, has given rise to many opinions. In addition, it would be possible to more accurately verify users’ considerations when they use Bitcoin through ‘security’ concepts relative to the problems that may occur as a result of mining and transactions.

The ‘silkroad’, a large marketplace that uses Bitcoin as a currency, has been exploited for illegal transactions and money laundering. Security therefore not only became a popular issue on the Bitcoin forum but also resulted in social problems, leading to the closure of the site. Although the situation was resolved when the site was closed towards the end of 2013, words regarding related exchange markets and companies attracted considerable attention from users. Therefore, many opinions on illegality related with the use of Bitcoin and consequent problems were verified through the concept of ‘illegality’.

Since the emergence of Bitcoin, many types of similar cryptocurrencies have been developed and are in use. Users’ discussion on the presence and availability of other cryptocurrencies can be found through the ‘altcoin’ concept. China dominates the pricing of Bitcoin with large funds, of which the trend manifest in the postings on the forum can be viewed via the concept ‘China’.

### Results of Granger causality test and correlation test

In view of the after-effects of the Granger causality test, the null hypothesis was rejected. This suggests that the time series of the gathered data failed to forecast the fluctuation in Bitcoin transaction volume and price—i.e. *β*_{1,2,⋯,*b*}_ ≠ 0—with a high level of confidence. The Granger causality test was performed on the Bitcoin transaction count and price for a time lag of 1 to 12 days. Tables 3 and 4 list the test results.

**Table 3:** Statistical significance (*p*-values) of bivariate Granger causality correlation between Bitcoin price and concepts of forum opinions.

Time Lag	Bitcoin Price									
	Altcoin	Blockchain	China	Illegal	Investment	Mining	Security	Silkroad	Transaction	Wallet
1 day	0.6204	0.3299	0.0914	0.5226	0.6678	0.4277	0.6371	0.8107	0.7004	0.3202
2 days	0.5679	0.4181	**0.0011**	0.4320	0.6507	0.3697	0.6519	0.6195	0.1951	0.3786
3 days	0.7593	0.3582	**0.0005**	0.3818	0.7483	0.4542	0.8595	0.391	0.3279	0.388
4 days	0.7755	0.2349	**0.0005**	0.413	0.6438	0.5089	0.5715	0.6442	0.2472	0.4215
5 days	0.8983	0.4554	**0.0017**	0.4653	0.821	0.568	0.5784	0.5936	0.3554	0.5256
6 days	0.8783	0.8423	**0.000001**	0.5382	0.9669	0.5894	0.792	0.5116	0.3812	0.7491
7 days	0.8763	0.8863	**0.000002**	0.5424	0.8627	0.616	0.4688	0.3092	0.3932	0.784
8 days	0.8582	0.9234	**0.000003**	0.5537	0.9132	0.633	0.5301	0.3558	0.4583	0.9324
9 days	0.8369	0.8167	**0.00007**	0.5666	0.9492	0.4762	0.3901	0.2966	0.4029	0.9656
10 days	0.7949	0.7043	**0.0018**	0.6014	0.9504	0.474	0.3984	0.1223	0.2574	0.9796
11 days	0.8005	0.6991	**0.0023**	0.5934	0.9305	0.4884	0.3482	0.1093	0.2409	0.9954
12 days	0.6838	0.5004	**0.0006**	0.5916	0.9381	0.2453	0.0473	0.095	0.329	0.9753

**Table 4:** Statistical significance (*p*-values) of bivariate Granger causality correlation between Bitcoin transaction and concept of forum opinions.

Time Lag	Bitcoin Number of Transactions									
	Altcoin	Blockchain	China	Illegal	Investment	Mining	Security	Silkroad	Transaction	Wallet
1 day	0.4827	**0.0142**	0.9888	0.0915	**0.005**	**0.0483**	0.5636	0.4659	**0.0195**	**0.0493**
2 days	0.5696	**0.0487**	0.1418	0.1997	**0.0048**	0.1803	0.3015	**0.05**	0.0595	0.0909
3 days	0.6731	0.1271	0.1130	0.2961	**0.0043**	0.1041	0.5669	0.0880	0.1005	0.1152
4 days	**0.0160**	0.0625	0.2018	0.4679	**0.0273**	0.2037	0.6715	0.1431	0.0705	0.2638
5 days	**0.0450**	**0.0004**	0.22	0.3464	0.2432	0.3233	0.4783	0.1845	**0.003**	0.5231
6 days	**0.0023**	**0.0001**	0.2252	0.1459	0.5689	0.2395	0.4350	0.2226	**0.0042**	0.7785
7 days	**0.0009**	**0.00003**	0.3597	0.1456	0.8021	0.3042	0.4575	0.3356	**0.0054**	0.8747
8 days	**0.0011**	**0.0004**	0.4325	0.1632	0.7045	0.4271	0.4080	0.3630	**0.0074**	0.949
9 days	**0.0004**	**0.0008**	0.5079	0.0844	0.7806	0.4939	0.4236	0.337	**0.0102**	0.9743
10 days	**0.0008**	**0.0013**	0.5734	0.056	0.5875	0.3762	0.5061	0.4175	**0.0165**	0.8722
11 days	**0.00001**	**0.0024**	0.6412	0.0589	0.6785	0.4631	0.6185	0.5021	**0.0185**	0.9269
12 days	**0.00002**	**0.0040**	0.7429	**0.05**	0.7327	0.5322	0.6857	0.4687	**0.0277**	0.9476

In addition, the Pearson Correlation Coefficient between the rating of each concept and Bitcoin price and transaction is shown in Table 5.

**Table 5:** Pearson Correlation Coefficient result.

Concept	Bitcoin Price	Bitcoin Number of Transactions
Mining	0.3384	0.2353
Transaction	0.071	0.0424
Silkroad	-0.1189	-0.1139
Illegal	0.2026	0.1523
Blockchain	-0.0207	-0.0566
Altcoin	0.6394	0.4553
Wallet	0.436	0.3581
China	0.1556	0.3115
Security	0.0883	0.0564
Investment	0.5949	0.4177

The foregoing results are partially indicative of the significance of the extracted keyword data. However, this process was only used for the purpose of verification. The entire data set was used to build the actual deep learning model for prediction.

### Prediction results

We built and applied the deep learning model based on the gathered and KDE-based rating data to predict the Bitcoin transaction and price.

For the period from 1 December 2013 to 21 September 2016, 90% of the data were used for learning, with the remaining 10% used for validation. The accuracy rate, the Matthews correlation coefficient (MCC), and the F-measure were used to evaluate the performance of the proposed model.

Table 6 presents the prediction results. The most accurate prediction model for the Bitcoin price (accuracy rate = 80.39%) is based on the three-layer neural network and the previous twelve-day learning data. The most accurate prediction model for Bitcoin transaction (accuracy rate = 81.37%) is based on the two-layer neural network and the previous twelve-day learning data. Table 4 presents the results relative to the layer and learning data structures. Both three or more hidden layers and cumulative learning data for 12 days or longer resulted in negligible differences. Less than two hidden layers and cumulative learning data for less than 7 days proved to be insufficient for learning and compromised the prediction accuracy. Conversely, overfitting could possibly occur with the prediction accuracy failing to significantly improve, if more than five hidden layers and cumulative data for over 12 days were used.

**Table 6:** Experimental results of predicted Bitcoin fluctuation.

Data Set		Bitcoin Price			Bitcoin Number of Transactions		
Hidden Layers	Learning Days	Accuracy (%)	F1-Score	MCC	Accuracy (%)	F1-Score	MCC
1 Hidden Layer	3 Days	55.88%	0.559	0.1185	62.75%	0.6834	0.3911
	5 Days	60.78%	0.6074	0.2134	68.63%	0.7319	0.4492
	7 Days	63.73%	0.6374	0.274	78.43%	0.8143	0.5504
	12 Days	65.69%	0.6567	0.3122	74.51%	0.7929	0.4608
2 Hidden Layers	3 Days	56.86%	0.5688	0.1374	64.7%	0.776	0.1319
	5 Days	58.82%	0.5854	0.1902	70.59%	0.8181	0.1506
	7 Days	64.7%	0.6432	0.3147	**81.37%**	**0.8346**	**0.5355**
	12 Days	70.59%	0.7054	0.4203	75.5%	0.7684	0.379
3 Hidden Layers	3 Days	65.69%	0.6599	0.3087	66.67%	0.669	0.314
	5 Days	69.6%	0.7001	0.3772	68.63%	0.6885	0.3546
	7 Days	74.51%	0.7488	0.4659	75.5%	0.7553	0.4959
	12 Days	**80.39%**	**0.8076**	**0.5771**	79.41%	0.7945	0.5766
5 Hidden Layers	3 Days	63.73%	0.639	0.2771	64.7%	0.6438	0.308
	5 Days	65.69%	0.658	0.3183	67.65%	0.6734	0.3561
	7 Days	67.65%	0.6767	0.3593	71.57%	0.7136	0.4223
	12 Days	72.55%	0.746	0.5439	70.59%	0.704	0.3992

## Discussion

We analysed the user comments posted on a Bitcoin online forum to predict the fluctuation in the Bitcoin price and transaction count. Based on the easily accessible online data, the proposed method predicted the Bitcoin price fluctuation with an accuracy rate of over 80%. Moreover, online user postings influenced Bitcoin transactions. The proposed method shed light on some aspects of Bitcoin-related user comments affecting their decisions to buy/sell the cryptocurrency.

The causality test result indicated some topics associated with Bitcoin transactions. The Granger causality test result highlighted the concept ‘China’ as having a high causality toward the Bitcoin price with the p-value being 0.05 or less, which was significant. These findings suggest China exerts a strong influence on the Bitcoin price.

Furthermore, such concepts as Blockchain’, ‘Altcoin’, and ‘Transaction’ had a high causality toward Bitcoin transaction count with the p-value being 0.05 or less, which was significant. This finding suggests that topics related to the circulation and transaction of other types of cryptocurrencies have an impact on the Bitcoin transaction volume.

In addition, the correlation test found significant linear relations in most concepts, excluding ‘Silkroad’, which showed an insignificant linear relation. Hence, the experimental findings revealed some user comments that had the most significant relationship with and effects on the fluctuation in Bitcoin price and transactions.

That said, the proposed method has a limitation in terms of its broader applicability due to the fact that the concepts were constructed for a long period of time. For instance, the correlation coefficient of the concept ‘Silkroad’ was 0 or lower even though its construction was based on topics often mentioned by users in relation to some events taking place during a certain period, which hindered the extension of the analysis of the concept to the entire sample period. Thus, appropriate subdivision of the sample period would help to obtain a more accurate understanding of the users for topic modelling and to refine the analysis with additional approaches including sentiment analysis.

Moreover, the present findings warrant further studies on the analysis of user comments relative to the characteristics of Bitcoin forums.

To increase the accuracy of prediction, it is necessary to address a few challenges. The present work is focused on analysing online forum user comments and adds some formal or structured data to predict the fluctuation in the Bitcoin price and transactions. However, it may add to the reliability of the findings if the search results and relevant content on search engines were quantitatively analysed or if the social network data were analysed as they did in some comparable previous studies[21, 40]. Furthermore, it may be an efficient preliminary study to analyse and classify online forum users per se[41–45]. In addition, the postings may be worth filtering more meticulously [46–50] to more accurately corroborate the findings.

Information derived from online forum users seems to be well-suited for extensive research on cryptocurrencies as well as Bitcoin. In the same vein, keywords manifested in online forum user comments could be used for further in-depth analysis and understanding of cryptocurrency transactions. Online forum users’ propensities could also be a cue to identify the characteristics inherent in each cryptocurrency. Moreover, online forums are great sources of abundant informal and formal information, which serves to appreciate cryptocurrencies from diverse perspectives including money laundering, which is closely associated with cryptocurrencies [51–54].

## Conclusion

With the increasing circulation of Bitcoin, its acceptability has drawn much attention in many ways [2, 3, 5, 14]. The present study is noteworthy in that it analysed the topics often mentioned by Bitcoin users and linked their meanings to Bitcoin transactions. The proposed method for predicting the fluctuation in the Bitcoin price and transactions based on user opinions on online forums is conducive to understanding a range of cryptocurrencies other than Bitcoin and increasing their usability, although it needs to be reinforced. In addition, the present approach to the salience of user comments on online forums is likely to yield more significant results in many other fields.

## Supporting information

S1 File Results of crawling Bitcoin forum. (ZIP)

S2 File Python-based crawler source code for Bitcoin forum data collection. (ZIP)

## References

[1] Nakamoto S. Bitcoin: A peer-to-peer electronic cash system. 2008.

[2] Böhme R, Christin N, Edelman B, Moore T. Bitcoin: Economics, technology, and governance. The Journal of Economic Perspectives. 2015;29(2):213–38.

[3] Grinberg R. Bitcoin: An innovative alternative digital currency. Hastings Sci & Tech LJ. 2012;4:159.

[4] Barber S, Boyen X, Shi E, Uzun E. Bitter to better—how to make bitcoin a better currency. International Conference on Financial Cryptography and Data Security. 2012:399–414.

[5] Reid F, Harrigan M. An analysis of anonymity in the bitcoin system. Security and privacy in social networks. 2013:197–223.

[6] Kim YB, Kim JG, Kim W, Im JH, Kim TH, Kang SJ, et al Predicting Fluctuations in Cryptocurrency Transactions Based on User Comments and Replies. PLOS ONE. 2016;11(8):e0161197 doi: 10.1371/journal.pone.0161197 27533113 10.1371/journal.pone.0161197 PMC4988639

[7] Abadi M, Agarwal A, Barham P, Brevdo E, Chen Z, Citro C, et al. Tensorflow: Large-scale machine learning on heterogeneous distributed systems. arXiv preprint arXiv:160304467. 2016.

[8] LeCun Y, Bengio Y, Hinton G. Deep learning. Nature. 2015;521(7553):436–44. doi: 10.1038/nature14539 26017442 10.1038/nature14539

[9] Matta M, Lunesu I, Marchesi M, editors. Bitcoin Spread Prediction Using Social and Web Search Media. UMAP Workshops; 2015.

[10] Kaminski J. Nowcasting the Bitcoin Market with Twitter Signals. arXiv preprint arXiv:14067577. 2014.

[11] Kristoufek L. BitCoin meets Google Trends and Wikipedia: Quantifying the relationship between phenomena of the Internet era. Scientific reports. 2013;3. 10.1038/srep03415 PMC3849639 24301322

[12] Kristoufek L. What are the main drivers of the Bitcoin price? Evidence from wavelet coherence analysis. PloS one. 2015;10(4):e0123923 doi: 10.1371/journal.pone.0123923 25874694 10.1371/journal.pone.0123923 PMC4398561

[13] Yelowitz A, Wilson M. Characteristics of Bitcoin users: an analysis of Google search data. Applied Economics Letters. 2015;22(13):1030–6.

[14] Athey S, Parashkevov I, Sarukkai V, Xia J. Bitcoin pricing, adoption, and usage: Theory and evidence. 2016.

[15] Bernstein MS, Monroy-Hernández A, Harry D, André P, Panovich K, Vargas GG. 4chan and/b: An Analysis of Anonymity and Ephemerality in a Large Online Community. ICWSM. 2011:50–7.

[16] Hau YS, Kim Y-G. Why would online gamers share their innovation-conducive knowledge in the online game user community? Integrating individual motivations and social capital perspectives. Computers in Human Behavior. 2011;27(2):956–70.

[17] Kim YB, Lee SH, Kang SJ, Choi MJ, Lee J, Kim CH. Virtual world currency value fluctuation prediction system based on user sentiment analysis. PloS one. 2015;10(8):e0132944 doi: 10.1371/journal.pone.0132944 26241496 10.1371/journal.pone.0132944 PMC4524693

[18] Panzarasa P, Opsahl T, Carley KM. Patterns and dynamics of users' behavior and interaction: Network analysis of an online community. Journal of the American Society for Information Science and Technology. 2009;60(5):911–32.

[19] Sing CC, Khine MS. An analysis of interaction and participation patterns in online community. JOURNAL OF EDUCATIONAL TECHNOLOGYAND SOCIETY. 2006;9(1):250.

[20] Fleder M, Kester MS, Pillai S. Bitcoin transaction graph analysis. arXiv preprint arXiv:150201657. 2015.

[21] Cohen-Charash Y, Scherbaum CA, Kammeyer-Mueller JD, Staw BM. Mood and the market: can press reports of investors' mood predict stock prices? PloS one. 2013;8(8):e72031 doi: 10.1371/journal.pone.0072031 24015202 10.1371/journal.pone.0072031 PMC3756040

[22] Bollen J, Mao H, Zeng X. Twitter mood predicts the stock market. Journal of Computational Science. 2011;2(1):1–8.

[23] Linton M, Teo EG, Bommes E, Chen CY-H, Härdle WK. Dynamic Topic Modelling for Cryptocurrency Community Forums. 2016.

[24] Blei DM, Ng AY, Jordan MI. Latent dirichlet allocation. Journal of machine Learning research. 2003;3(Jan):993–1022.

[25] Lee DD, Seung HS. Learning the parts of objects by non-negative matrix factorization. Nature. 1999;401(6755):788–91. doi: 10.1038/44565 10548103 10.1038/44565

[26] Wang C, Blei DM. Collaborative topic modeling for recommending scientific articles. Proceedings of the 17th ACM SIGKDD international conference on Knowledge discovery and data mining. 2011:448–56.

[27] Wang Y, Agichtein E, Benzi M. TM-LDA: efficient online modeling of latent topic transitions in social media. Proceedings of the 18th ACM SIGKDD international conference on Knowledge discovery and data mining. 2012:123–31.

[28] Xianghua F, Guo L, Yanyan G, Zhiqiang W. Multi-aspect sentiment analysis for Chinese online social reviews based on topic modeling and HowNet lexicon. Knowledge-Based Systems. 2013;37:186–95.

[29] Bohr J, Bashir M. Who uses bitcoin? an exploration of the bitcoin community. Privacy, Security and Trust (PST), 2014 Twelfth Annual International Conference on. 2014:94–101.

[30] Choi H, Varian H. Predicting the present with Google Trends. Economic Record. 2012;88(s1):2–9.

[31] Preis T, Moat HS, Stanley HE. Quantifying trading behavior in financial markets using Google Trends. Scientific reports. 2013;3. 10.1038/srep01684 PMC3635219 23619126

[32] Kristoufek L. Can Google Trends search queries contribute to risk diversification? Scientific reports. 2013;3. 10.1038/srep02713 PMC3776958 24048448

[33] Kang M, Zhong H, He J, Rutherford S, Yang F. Using google trends for influenza surveillance in South China. PloS one. 2013;8(1):e55205 doi: 10.1371/journal.pone.0055205 23372837 10.1371/journal.pone.0055205 PMC3555864

[34] Kim YB, Park N, Zhang Q, Kim JG, Kang SJ, Kim CH. Predicting Virtual World User Population Fluctuations with Deep Learning. PLOS ONE. 2016;11(12):e0167153 doi: 10.1371/journal.pone.0167153 27936009 10.1371/journal.pone.0167153 PMC5147861

[35] Moat HS, Curme C, Avakian A, Kenett DY, Stanley HE, Preis T. Quantifying Wikipedia usage patterns before stock market moves. Scientific reports. 2013;3.

[36] Mestyán M, Yasseri T, Kertész J. Early prediction of movie box office success based on Wikipedia activity big data. PloS one. 2013;8(8):e71226 doi: 10.1371/journal.pone.0071226 23990938 10.1371/journal.pone.0071226 PMC3749192

[37] Mikolov T, Chen K, Corrado G, Dean J. Efficient estimation of word representations in vector space. arXiv preprint arXiv:13013781. 2013.

[38] Granger CW, Huangb B-N, Yang C-W. A bivariate causality between stock prices and exchange rates: evidence from recent Asianflu☆. The Quarterly Review of Economics and Finance. 2000;40(3):337–54.

[39] Schmidhuber J. Deep learning in neural networks: An overview. Neural Networks. 2015;61:85–117. doi: 10.1016/j.neunet.2014.09.003 25462637 10.1016/j.neunet.2014.09.003

[40] Matta M, Lunesu I, Marchesi M. Bitcoin spread prediction using social and web search media. Proceedings of DeCAT. 2015.

[41] Juea W, Jian-pinga Z, Bao-huab Z, Cheng-ronga W. Online Forum Opinion Leaders Discovering Method Based on Clustering Analysis [J]. Computer Engineering. 2011;5:017.

[42] Li N, Wu DD. Using text mining and sentiment analysis for online forums hotspot detection and forecast. Decision support systems. 2010;48(2):354–68.

[43] Coles BA, West M. Trolling the trolls: Online forum users constructions of the nature and properties of trolling. Computers in Human Behavior. 2016;60:233–44.

[44] Benevenuto F, Rodrigues T, Cha M, Almeida V. Characterizing user behavior in online social networks. Proceedings of the 9th ACM SIGCOMM conference on Internet measurement conference. 2009:49–62.

[45] Maia M, Almeida J, Almeida V. Identifying user behavior in online social networks. Proceedings of the 1st workshop on Social network systems. 2008:1–6.

[46] Rout J, Dalmia A, Choo K-KR, Bakshi S, Jena S. Revisiting Semi-supervised Learning for Online Deceptive Review Detection. IEEE Access. 2017.

[47] Feng S, Banerjee R, Choi Y, editors. Syntactic stylometry for deception detection. Proceedings of the 50th Annual Meeting of the Association for Computational Linguistics: Short Papers-Volume 2; 2012: Association for Computational Linguistics.

[48] Shin Y, Gupta M, Myers S, editors. Prevalence and mitigation of forum spamming. INFOCOM, 2011 Proceedings IEEE; 2011: IEEE.

[49] Shin Y, Gupta M, Myers SA, editors. The Nuts and Bolts of a Forum Spam Automator. LEET; 2011.

[50] Hayati P, Potdar V, editors. Toward spam 2.0: an evaluation of web 2.0 anti-spam methods. Industrial Informatics, 2009 INDIN 2009 7th IEEE International Conference on; 2009: IEEE.

[51] Choo K-KR. Designated non-financial businesses and professionals: A review and analysis of recent financial action task force on money laundering mutual evaluation reports. Security Journal. 2014;27(1):1–26.

[52] Choo K-KR. Cryptocurrency and Virtual Currency: Corruption and Money Laundering/Terrorism Financing Risks? Handbook of Digital Currency: Bitcoin, Innovation, Financial Instruments, and Big Data. 2015:283.

[53] Bryans D. Bitcoin and money laundering: mining for an effective solution. Ind LJ. 2014;89:441.

[54] Stokes R. Virtual money laundering: the case of Bitcoin and the Linden dollar. Information & Communications Technology Law. 2012;21(3):221–36.

